# Expression and clinical significance of FANCI gene in pan-cancer: a comprehensive analysis based on multi-omics data

**DOI:** 10.3389/fgene.2025.1542888

**Published:** 2025-05-09

**Authors:** Yunzheng Zhao, Qingyu Li, Jiajun Li, YiFeng Cui, Zhaoyang Lu

**Affiliations:** ^1^ Department of Hepatic Surgery, The First Affiliated Hospital of Harbin Medical University, Harbin, China; ^2^ Key Laboratory of Hepatosplenic Surgery, Ministry of Education, The First Affiliated Hospital of Harbin Medical University, Harbin, China

**Keywords:** FANCI, pan-cancer, DNA damage repair, gene expression, clinical significance, biomarker

## Abstract

**Introduction:**

The FANCI gene, an essential element of the Fanconi anemia pathway, has been associated with a variety of cancer types. This investigation seeks to clarify the expression profiles, prognostic relevance, and diagnostic capabilities of FANCI across multiple malignancies, along with its links to immune cell infiltration, genetic alterations, protein-protein interactions, and functional roles.

**Methods:**

By utilizing data from The Cancer Genome Atlas (TCGA) and Genotype-Tissue Expression (GTEx) databases, we conducted a comprehensive analysis of FANCI mRNA expression using R software and visualized the results with the ggplot2 package. Prognostic and diagnostic evaluations were conducted using Xiantao tools to produce survival and receiver operating characteristic (ROC) curves. The examination of genetic variation was facilitated through cBioPortal, while DNA methylation and mRNA modifications were analyzed utilizing UALCAN and SangerBox 3.0. Correlations with immune responses were assessed via the EPIC platform and SangerBox 3.0. Additionally, we constructed protein-protein interaction networks employing the STRING database and Cytoscape software. Functional enrichment analyses encompassed Gene Ontology (GO), Kyoto Encyclopedia of Genes and Genomes (KEGG), and Gene Set Enrichment Analysis (GSEA). The CancerSEA database was also utilized for single-cell level investigation of FANCI’s association with the functional states of cancer.

**Results:**

Our findings reveal that FANCI is significantly upregulated in the majority of tumor types when compared to normal tissues, with increased protein levels observed in several cancers, including colorectal adenocarcinoma (COAD) and pancreatic adenocarcinoma (PAAD). Elevated FANCI expression is associated with unfavorable prognoses in cancers such as adrenocortical carcinoma (ACC) and liver hepatocellular carcinoma (LIHC). Methylation assessments demonstrated a robust inverse correlation between FANCI promoter methylation and its expression in LIHC. Moreover, FANCI expression was found to be connected to immune cell infiltration and tumor mutation burden in select cancers.

**Discussion:**

In summary, FANCI presents as a promising biomarker for cancer prognosis and diagnosis, with potential implications for therapeutic interventions. Subsequent investigations should concentrate on elucidating the mechanistic functions of FANCI in cancer development and assessing its viability as a therapeutic target.

## Introduction

Cancer remains a leading cause of death worldwide, with its burden escalating due to demographic changes and persistent risk factors. According to the Global Cancer Observatory (GLOBOCAN 2023), an estimated 20.3 million new cancer cases and 9.7 million cancer-related deaths occurred globally in 2023, reflecting a 4% increase in incidence and 2% rise in mortality compared to 2020 estimates (Bray et al., 2024; Inzani and Rindi, 2021). The complexity and heterogeneity of the disease, driven by a combination of genetic and environmental factors, present significant challenges for effective treatment and management. Despite advancements in cancer research, the prognosis for many cancer types remains unfavorable, underscoring the urgent need for the identification of novel biomarkers and therapeutic targets (Wang et al., 2024; Wang et al., 2019). Recent advances in multi-omics technologies and artificial intelligence-driven biomarker discovery have identified promising targets, such as clonal neoantigens and metabolic vulnerabilities (Sharma et al., 2017; Lu et al., 2021). Nevertheless, over 60% of advanced solid tumors still develop resistance to first-line therapies, highlighting critical gaps in precision oncology (Arora et al., 2023). Urgent efforts are needed to unravel context-specific oncogenic mechanisms and translate molecular insights into durable clinical responses.

FANCI (Fanconi anemia complementation group I) is a critical gene involved in the DNA damage response and repair pathways, particularly within the Fanconi anemia (FA) pathway (Smogorzewska et al., 2007; Sareen et al., 2012; Koczorowska et al., 2014). FANCI, in conjunction with FANCD2, forms a complex that is monoubiquitinated in response to DNA damage, facilitating the repair of interstrand crosslinks (Rego et al., 2012a; Lopez-Martinez et al., 2019). Dysregulation of the FA pathway, including mutations or altered expression of FANCI, has been linked to genomic instability and increased cancer susceptibility (Altintas et al., 2023).

Previous studies have highlighted the involvement of FANCI in various cancer types. For example, FANCI mutations have been identified in ovarian and breast cancers, suggesting a role in tumorigenesis (Birkbak et al., 2018; Fierheller et al., 2021). In colorectal cancer, altered FANCI expression has been associated with poor prognosis and resistance to chemotherapy (Xu et al., 2021). In lung cancer, FANCI downregulation has been correlated with increased sensitivity to DNA-damaging agents, indicating its potential as a therapeutic target (Li et al., 2023).

Given the diverse roles of FANCI across different cancers, a comprehensive pan-cancer analysis is warranted to elucidate its expression patterns, prognostic significance, and potential as a diagnostic biomarker. This study aims to investigate FANCI expression across multiple cancer types using data from The Cancer Genome Atlas (TCGA) and Genotype-Tissue Expression (GTEx) databases. Statistical analyses and visualization tools, including R software and ggplot2, will be employed to assess FANCI expression levels and their association with clinical outcomes.

Furthermore, we will explore FANCI’s genetic alterations using cBioPortal, examining mutation types and copy number variations. DNA methylation and mRNA modification patterns will be analyzed using UALCAN and SangerBox 3.0 tools. The relationship between FANCI expression and immune cell infiltration will be evaluated via the EPIC platform, while its association with tumor mutation burden will be investigated using SangerBox 3.0. Protein-protein interaction networks will be constructed using STRING and Cytoscape software to identify key interacting partners and functional modules. Functional enrichment analyses, including Gene Ontology (GO) and Kyoto Encyclopedia of Genes and Genomes (KEGG) pathways, will be conducted to uncover biological processes and pathways associated with FANCI. Additionally, we will utilize the CancerSEA database to study FANCI’s correlation with cancer functional states at the single-cell level. Finally, a regulatory network involving long non-coding RNAs (lncRNAs), microRNAs (miRNAs), and FANCI will be constructed using various online databases and StarBase 2.0.

In summary, this study aims to provide a comprehensive understanding of FANCI’s role in cancer, highlighting its potential as a biomarker for prognosis and diagnosis, and uncovering novel therapeutic targets. By integrating multi-omics data and employing robust bioinformatics analyses, we hope to elucidate the complex interactions and regulatory mechanisms involving FANCI in different cancer contexts.

## Materials and methods

### Expression and subcellular localization analysis of FANCI

FANCI mRNA expression levels in tumor, adjacent paracancerous, and normal tissues were obtained from TCGA and GTEx databases (Vivian et al., 2017) Samples with “0” expression were excluded, and paired samples were used for comparison. RNA sequencing data in FPKM were normalized to TPM using the Toil pipeline and log2 transformed. Statistical analyses were conducted with R software (v3.6.3), using “ggplot2” (v3.3.3) for bar charts of FANCI expression across 33 cancer types. Median expression values set cutoff thresholds, and the Wilcoxon rank-sum test assessed intergroup differences. Subcellular localization of FANCI was visualized in A-431 and U251MG cell lines using immunofluorescence from the Human Protein Atlas (HPA), with immunohistochemistry images also sourced from HPA.

### Evaluation of FANCI’s prognostic and diagnostic significance

We utilized TCGA data and conducted COX regression analysis using the Xiantao tool, considering a P-value less than 0.05 as statistically significant. Hazard ratios (HR) and 95% confidence intervals (CI) were derived from RNA sequencing data to assess the impact of FANCI expression on cancer diagnosis and prognosis. Results were visualized with a forest plot using the Xiantao tool. The diagnostic significance of FANCI across cancer types was evaluated with ROC curves and the area under the curve (AUC), categorizing diagnostic accuracy as low (AUC: 0.5–0.7), moderate (AUC: 0.7–0.9), and high (AUC >0.9).

### Gene alteration analysis

We used the cBioPortal for Cancer Genomics to analyze gene data from the TCGA Pan-Cancer Study, aiding in the interpretation of molecular data from cancer studies. Gene alteration data from 2,683 samples of 2,565 pan-cancer patients were obtained from UCSC Xena and the ICGC data portal. The “Cancer Types Summary” module explored the mutation landscape of FANCI, including mutation types, copy number alterations (CNA), and mutation frequency. Somatic mutation datasets were retrieved from the TCGA database and analyzed with the HOME bioinformatics tool.

### DNA methylation and mRNA modifications

UALCAN (http://ualcan.path.uab.edu/analysis.html) was employed to investigate the promoter DNA methylation levels of FANCI in normal and pan-cancer tissues. The β-value indicates DNA methylation levels, with low methylation defined as β: 0.3–0.25 and high methylation as β: 0.7–0.5. The DNA methylation profile of FANCI in liver hepatocellular carcinoma (LIHC) was obtained from MethSurv using the “Gene Visualization” module. For mRNA modification analysis, SangerBox 3.0 (http://sangerbox.com/) was used through the “Pan-Cancer Analysis-mRNA Modifications” module. This tool analyzed 45 methylation regulators involved in N1-methyladenosine (m1A), 5-methylcytosine (m5C), and N6-methyladenosine (m6A) across TCGA pan-cancer tissues.

### Correlation of FANCI expression with immunity

The EPIC platform provides scores based on stromal, immune, and ESTIMATE cell expression data in TCGA, detailing infiltration proportions for eight immune cell types. The LIHC dataset was exported using the “estimation” R package to evaluate tumor purity scores. The correlation between FANCI expression and eight immune checkpoints was explored using the “Gene_Corr” module, with “P-value” and “r-value” visualized via heatmaps in the Xiantao tool. Additionally, SangerBox 3.0’s “Pan-Cancer Analysis - Heterogeneity Analysis” module was used to investigate the relationship between FANCI expression and Tumor Mutation Burden (TMB).

### Protein-protein interaction (PPI) network analysis

The STRING database was used to gather potential protein interactions involving FANCI, with a confidence score threshold of 0.7. The data were imported into Cytoscape (v3.8.2) for visualization. The cytoHubba plugin identified key modules, with the top 10 nodes ranked by the MCC method designated as hub genes. Using the Pathlinker plugin, signaling pathways were reconstructed from these hub genes, calculating multiple short paths from receptors to transcription factors within the PPI network. We also explored correlations between hub genes across various cancers.

### Functional enrichment analysis of FANCI

We performed Gene Ontology (GO) and Kyoto Encyclopedia of Genes and Genomes (KEGG) enrichment analyses on genes related to FANCI using the STRING database with the clusterProfiler and org packages. The significance threshold was set at (p < 0.01). Results were visualized in bubble plots using ggplot2. Gene set enrichment analysis (GSEA) identified pathway differences between high and low FANCI expression groups, considering pathways significant with FDR <0.25 and adjusted (p < 0.05). Each analysis used 1,000 permutations. The top 15 enriched results were shown in a ridge plot, and GSEA results were illustrated with ggplot2 in R.

### Identification of FANC1-Regulating transcription factors

To predict the transcription factors regulating the FANC1 gene, this study employed bioinformatics analysis using NetworkAnalyst 3.0 (https://www.networkanalyst.ca/). The specific workflow is as follows: the gene symbol FANC1 was input into the platform, with the species set as *Homo sapiens*. Using the promoter region motifs (1,500 bp upstream of the transcription start site) from the JASPAR database, a position weight matrix (PWM) score >85% and FDR <0.05 were set as the screening thresholds, and the transcription factor-gene interaction network was visualized.

### CancerSEA

We used the CancerSEA database to assess FANCI’s functional roles across various cancer types at single-cell resolution. Our study focused on the average correlation between FANCI and cancer-related functional states, including invasion, metastasis, proliferation, EMT, angiogenesis, apoptosis, cell cycle progression, differentiation, DNA damage response, DNA repair, hypoxia, inflammation, quiescence, and stem cell-like properties. A correlation threshold of 0.3 and a p-value below 0.05 were set for FANCI’s associations with these states.

### Construction of the LncRNA - miRNA - FANCI regulatory network

The target miRNAs for FANCI were predicted using three online databases: DIANA-microT, miRWalk, and miRDB. StarBase 2.0 (https://starbase.sysu.edu.cn/) was utilized to analyze the lncRNA-miRNA interaction network and the correlation between miRNAs and FANCI (lncRNA). The filtering criteria included “strict specificity for mammals, humans, hg19, CLIP data with a threshold of ≥5, with or without degradation group data.” The interactions between miRNA and lncRNA, as well as the lncRNA-miRNA-FANCI network, were visualized using Sankey diagrams in the Xiangtan tool and Cytoscape, respectively.

### siRNA transfection

The siRNA was synthesized by HanBio. Transfection was conducted in the Huh7 cell line utilizing JetPRIME Transfection Reagent (Poly Plus, #114-15). The Huh7 cells were seeded in six-well plates at a density of 1.5 × 10^5^ cells per well and incubated at 37°C for 12 h. Subsequently, 50 nM siRNA and 4 μL jetPRIME were dissolved in 200 μL jetPRIME Buffer, left at room temperature for 10 min, and then added to the six-well plates. The culture medium was changed after 6 h of incubation. RNA and protein were extracted at 48 h and 72 h post-transfection, respectively, to assess knockdown efficiency. The siRNA sequences employed were as follows: siRNA NC primer: F: UUC​UCC​GAA​CGU​GUC​ACG​U, R: ACG​UGA​CAC​GUU​CGG​AGA​A. FANCI-si-1 primer F: GGG​GAU​UUG​CAG​AAA​GAA​ATT, R: UUU​CUU​UCU​GCA​AAU​CCC​CTT. FANCI-si-2 primer F: CAU​UGA​GCA​UGU​UCU​CCA​ATT, R: UUG​GAG​AAC​AUG​CUC​AAU​GTT. FANCI-si-3 primer F: GAA​GAA​CUU​UAA​AGU​UUU​ATT, R: UAA​AAC​UUU​AAA​GUU​CUU​CTT. FANCI-si-4 primer F: GAG​UCU​UGC​UAU​GGA​GAU​ATT, R: UAU​CUC​CAU​AGC​AAG​ACU​CTT.

### qPCR

RNA was extracted from cells using the Progema (#LS1040) RNA Extraction Kit. Operations began 48 h post-transfection: cells were washed three times with PBS, followed by the addition of 300 µL RNA lysis buffer and incubation at room temperature for 5 min. Next, 300 µL RNA dilution buffer was added and mixed thoroughly. The sample was centrifuged at 14,000 × g for 5 min. The supernatant was collected, mixed with 250 µL anhydrous ethanol, and promptly transferred to a spin column, followed by centrifugation at 14,000 × g for 1 min. After discarding the flow-through, the sample was washed twice with RNA wash buffer and subjected to a dry spin. Finally, 50 µL nuclease-free water was added to elute RNA.

The reverse transcription reaction was performed using the EnzyArtisan (#R102-02) Reverse Transcription Kit. A reaction system was prepared comprising 1 μg RNA, 4 µL 5× RT Buffer, and RNase-free ddH_2_O to a final volume of 20 µL. The reaction was carried out under the following conditions: 37°C for 15 min, followed by 85°C for 5 s, resulting in the synthesis of cDNA.

Subsequently, qPCR was conducted using the EnzyArtisan (#R204-01) qPCR MIX for relative quantification. The reaction mixture included 10 µL 2× S6 Universal SYBR qPCR Mix, 0.4 µL forward primer, 0.4 µL reverse primer, 1 µL cDNA, and RNase-free ddH_2_O to a final volume of 20 µL. The PCR program was set as follows: initial denaturation at 95°C for 30 s, followed by cycling at 95°C for 10 s and 60°C for 30 s per cycle, with the melting curve settings applied by the instrument’s default. Post-reaction, relative quantification was performed using the 2^−ΔΔCT^ method, with β-actin serving as the internal control. FANCI primer: F: CAC​CAC​ACT​TAC​AGC​CCT​TG, R: ATTCCTCCGGAGCTCTGAC.β-Actin primer F: AGA​GCT​ACG​AGC​TGC​CTG​AC, R: AGC​ACT​GTG​TTG​GCG​TAC​AG.

### Western blot

After 72 h of cell transfection, total cellular protein was extracted using RIPA lysis buffer (Beyotime, #P0013B) supplemented with protease inhibitor (Beyotime, #P1005). The cells were washed three times with PBS, then lysed with the lysis mixture, and collected using a cell scraper. The lysate was thoroughly mixed and centrifuged at 14,000×g for 5 min. A small portion of the supernatant was used to measure protein concentration with a BCA Protein Assay Kit (Beyotime, #P0012S), while the remaining supernatant was mixed with 5X SDS-PAGE protein loading buffer (Beyotime, #P0015), boiled at 100°C for 10 min, and stored for further use. Subsequently, the protein samples were separated using a 7.5% SDS-PAGE gel (Epizyme, #PG211) and transferred onto a PVDF membrane. The membrane was blocked with 5% skim milk for 1 h, followed by incubation with the primary antibody at 4°C overnight. After three washes with PBST, the membrane was incubated with the secondary antibody at room temperature for 1 h. Finally, the membrane was imaged using an infrared fluorescence scanner.

### Wound healing assay

After 72 h of transfection, cells were seeded into six-well plates at a density of 4 × 10^5^ cells per well. After incubating at 37°C for 12 h, a scratch was made using a 10 μL pipette tip. The cells were rinsed with PBS, and images were taken with a microscope to establish the baseline at 0 h. After an additional 24 h of incubation, the cells were imaged again. The extent of healing in the scratched areas was quantitatively assessed using ImageJ software.

### Transwell assay

Transwell chambers were pre-treated with Matrigel and incubated at 37°C for 3 h. 72 h after transfection, cells were resuspended in DMEM without FBS and seeded into the Transwell chambers (with and without Matrigel) at a density of 5 × 10^4^ cells per well. Simultaneously, DMEM containing FBS was added to the lower chamber. After incubating at 37°C for an additional 12 h, the cells were fixed with methanol and stained with 0.5% crystal violet. Cells that had not migrated through the insert were removed using a cotton swab, and the chambers were washed with PBS. Finally, the Transwell chambers were imaged under a microscope.

### Statistical analysis

Statistical analyses were conducted using the specified online database and R (R Studio version 1.2.1335, R version 3.6.3). For experimental data, GraphPad Prism 9.0 was used. Differences were assessed with Student’s t-test, and results are expressed as mean ± SD. Statistical significance was indicated by *(P < 0.05), **(P < 0.01), ***(P < 0.001), and ****(P < 0.0001).

## Results

### Comprehensive analysis of FANCI expression and localization in cancer and normal tissues

**FIGURE 1:**
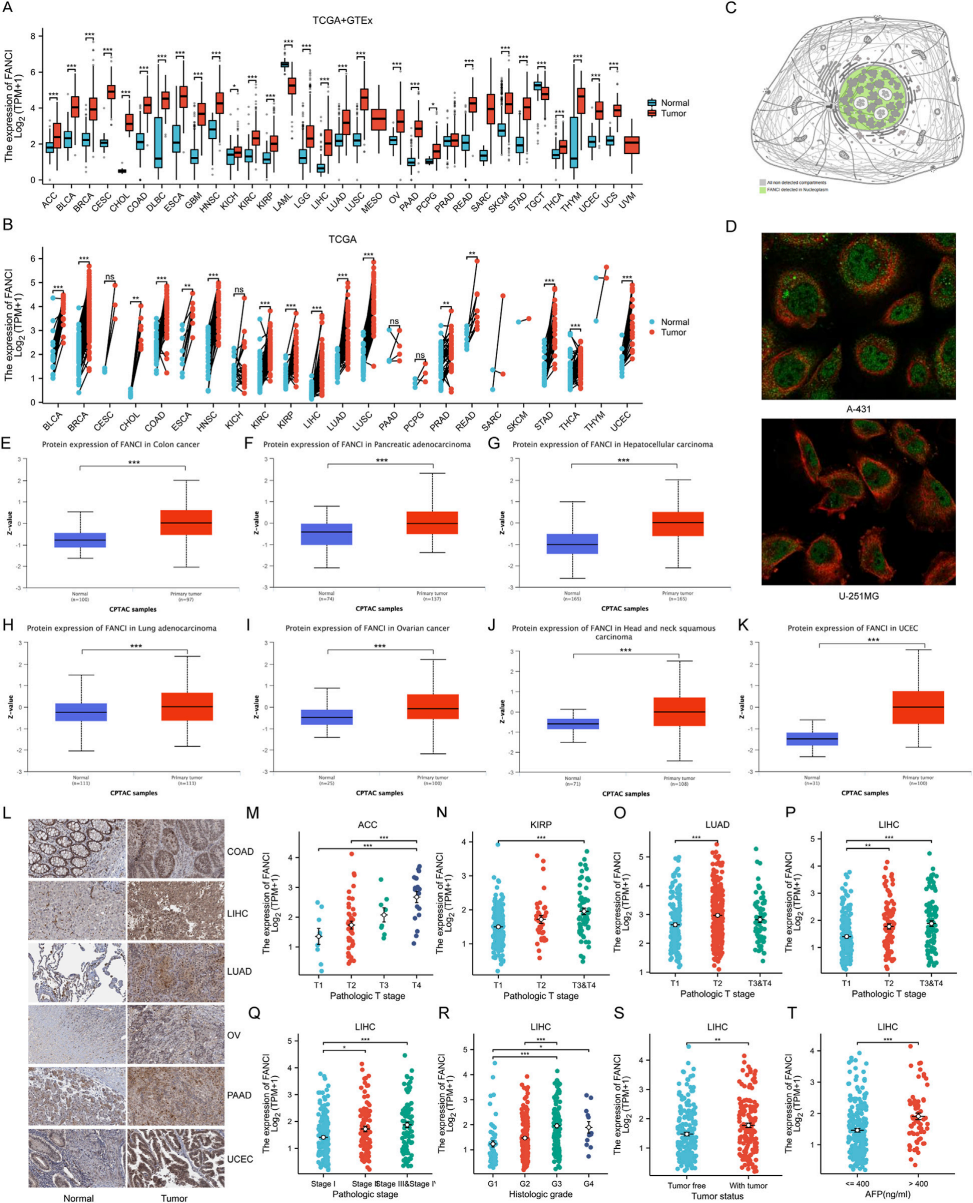
FANCI Expression Levels and Localization. **(A)** Comparison of FANCI mRNA expression in tumors versus normal tissues from TCGA and GTEx. **(B)** FANCI expression in tumors and paired adjacent normal tissues from TCGA (n = 15,043). **(C,D)** Subcellular localization of FANCI in A-431 and U-251MG cells from the HPA dataset, with green representing the target protein and red representing microtubules. **(E–K)** Comprehensive proteomic analysis showing FANCI expression across various cancers, providing a protein-level perspective. **(L)** Protein expression of FANCI in cancer and normal tissues detected by immunohistochemistry in the HPA dataset. **(M–T)** Correlations between FANCI expression and clinicopathological characteristics. *p < 0.05, **p < 0.01, ***p < 0.001.

The study presents an analysis of FANCI mRNA expression levels in tumor versus normal tissues using the TCGA+GTEx dataset. The box plot analysis reveals a significant upregulation of FANCI in the majority of tumor types compared to normal tissues, implicating a potential role for FANCI in tumorigenesis (Figure 1A). Further analysis of FANCI expression in tumors and adjacent normal tissues within the TCGA dataset corroborates these findings, with paired comparisons confirming the heightened expression of FANCI in tumor tissues (Figure 1B). Utilizing the HPA dataset, a schematic representation of FANCI’s subcellular localization indicates its predominant presence in the nucleus, aligning with its established function in DNA repair (Figure 1C). Immunofluorescence imaging of FANCI in A-431 and U-251MG cells underscores its nuclear localization, which is essential for its role in maintaining genome stability (Figure 1D).

The CPTAC dataset provides insights into FANCI protein expression across various cancer types, with box plots illustrating significantly elevated levels in COAD (Figure 1E), PAAD (Figure 1F), LIHC (Figure 1G), LUAD (Figure 1H), OV (Figure 1I), HNSC (Figure 1J), and UCEC (Figure 1K) relative to normal tissues. These protein expression findings complement the mRNA data, offering a holistic perspective on FANCI expression. Furthermore, immunohistochemical staining of selected cancer and corresponding normal tissues from the HPA dataset visually confirms the differential expression patterns observed in both mRNA and protein analyses, with more intense staining in tumor tissues (Figure 1L).

The study also examines the relationship between FANCI expression and various clinicopathological features. Correlations between FANCI expression and the pathological T stages of ACC, KIRP, LUAD, and LIHC are depicted (Figures 1M–P), revealing higher expression levels in advanced stages and significant associations with tumor staging and grading. A particular focus is given to LIHC, where FANCI expression markedly increases with advancing pathological stages (I-IV) of LIHC (Figure 1Q). Analysis of FANCI expression across different histological grades (G1-G4) of LIHC indicates significantly higher expression with increasing grades (Figure 1R). Comparisons of FANCI expression levels between tumor and non-tumor states of LIHC reveal significantly higher expression in the tumor group (Figure 1S). Additionally, FANCI expression at varying alpha-fetoprotein (AFP) levels (≤400 and >400 ng/mL) demonstrates significantly higher expression in the high AFP group (Figure 1T). Collectively, these findings suggest that FANCI expression is intricately linked to cancer progression and severity.

### Correlation of FANCI expression with prognosis and diagnosis across pan-cancer types

**FIGURE 2:**
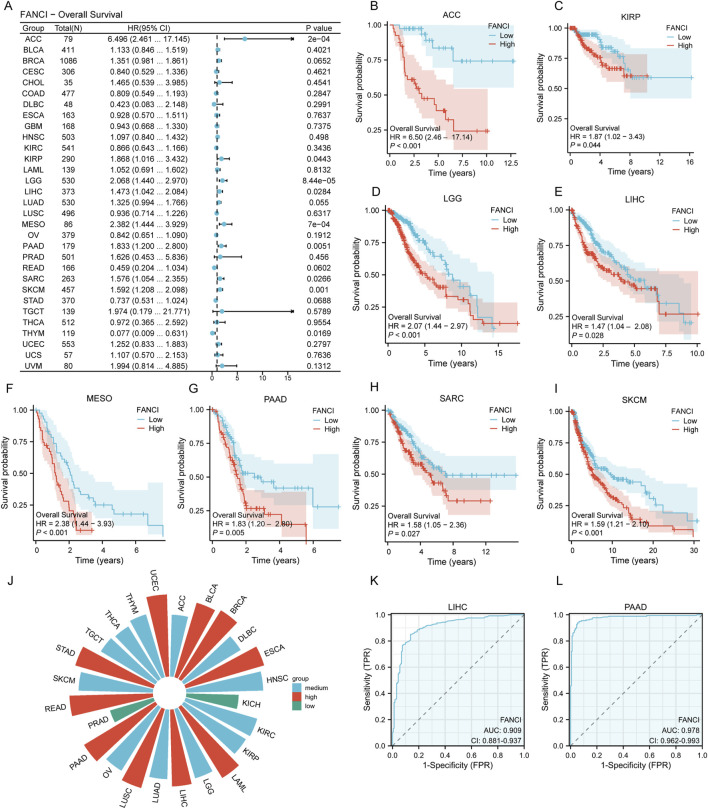
Correlation of FANCI Expression with Pan-Cancer Prognosis and Diagnosis. **(A)** Forest plot showing the association between FANCI expression and overall survival in various cancers, highlighting significant results (p < 0.05). **(B–I)** Kaplan-Meier survival curves illustrating the impact of FANCI expression on overall survival in specific cancers: ACC, KIRP, LGG, LIHC, MESO, PAAD, SARC, and SKCM. **(J)** ROC curve analysis assessing the diagnostic value of FANCI expression in pan-cancer, with AUC values indicating low (0.5-0.7), medium (0.7-0.9), and high (>0.9) accuracy. **(K,L)** ROC curves for LIHC (AUC = 0.909) and PAAD (AUC = 0.978).

Drawing from the forest plot and the Cox proportional hazards model analysis (Figure 2A), this study elucidates the relationship between FANCI expression and overall survival across various cancer types. The figure delineates the hazard ratios (HR) along with their 95% confidence intervals (CI). Notably, Kaplan-Meier survival curves for specific tumor types reveal that elevated FANCI expression is associated with decreased overall survival in several cancers, including ACC (Figure 2B, p = 0.0002), KIRP (Figure 2C, p = 0.0002), LGG (Figure 2D, p < 0.0001), LIHC (Figure 2E, p = 0.0284), MESO (Figure 2F, p = 0.0007), PAAD (Figure 2G, p = 0.0051), SARC (Figure 2H, p = 0.0266), and SKCM (Figure 2I, p = 0.0010). These survival curves suggest that high FANCI expression is significantly correlated with reduced overall survival in these cancer types.

Subsequently, receiver operating characteristic (ROC) curves were utilized to evaluate the discriminatory power of FANCI expression levels between malignant and non-tumor tissues. The area under the curve (AUC) was employed as the primary metric to assess the model’s discriminative capacity. In accordance with established conventions, AUC values ranging from 0.5 to 0.7 were interpreted as indicating low accuracy, from 0.7 to 0.9 as moderate accuracy, and above 0.9 as high accuracy. The results reveal that FANCI expression demonstrates substantial diagnostic potential in cancers such as BLCA, BRCA, ESCA, LAML, LIHC, LUSC, PAAD, READ, STAD, and UCEC (Figure 2J). Notably, among the cancers where high FANCI expression is linked to decreased overall survival, LIHC (Figure 2K, AUC = 0.909) and PAAD (Figure 2L, AUC = 0.978) exhibit significant diagnostic value. These findings suggest that FANCI expression could serve as a promising biomarker for cancer diagnosis.

### Genetic alterations and methylation patterns of FANCI in cancers

In our study, we employed the cBioPortal tool to investigate the pan-cancer genetic alterations of the FANCI gene. The gene mutation profile revealed that FANCI was altered in 2.7% of the samples (Supplementary Figure S1A), with the most prevalent types being “amplification,” represented by red bars, and “missense mutations,” depicted by green bars. Subsequently, we examined the mutation frequency of FANCI across 32 cancer types, as illustrated in Supplementary Figure S1B. The highest mutation rate was observed in endometrial cancer, exceeding 8%, predominantly characterized by the “mutation” type. Gastric adenocarcinoma ranked second, with a mutation rate surpassing 5%, where the “amplification” type was most common. Melanoma followed closely, also exceeding a 5% mutation rate, with the “mutation” type being the most frequent. Lastly, as shown in Supplementary Figure S1C, we explored the correlation between presumed copy number alterations (CNAs) of FANCI and its mRNA expression across pan-cancer tissues.

**FIGURE 3:**
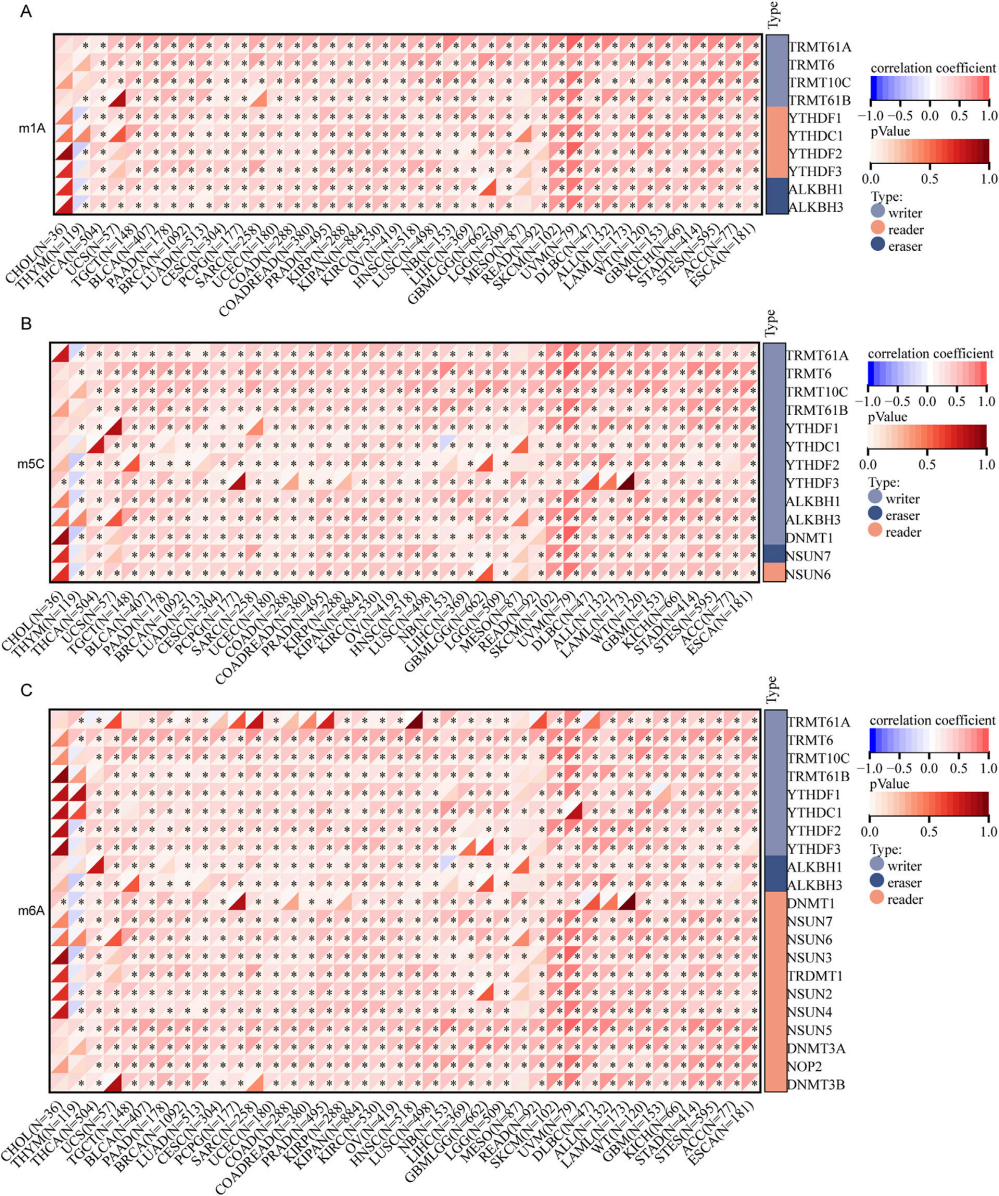
Analysis of the correlation between FANCI expression and regulatory factors involved in mRNA modification methylation: **(A)** m1A, **(B)** m5C, **(C)** m6A. The associations are evaluated using Pearson’s correlation coefficient and assessed for statistical significance (*p < 0.05).

In addition, we examined the methylation patterns of FANCI in cancer, as aberrant DNA methylation is associated with gene dysregulation in oncogenesis. A substantial body of research has demonstrated a close association between mRNA modifications and the progression and pathogenesis of cancer, implicating these modifications in post-transcriptional gene regulation. Among the prevalent types of mRNA modifications are N1-methyladenosine (m1A), 5-methylcytosine (m5C), and N6-methyladenosine (m6A). Our investigation commenced with an analysis of the correlation between FANCI expression and 45 mRNA modification regulatory factors, including methyltransferases (writers), demethylases (erasers), and RNA-binding proteins (readers). As depicted in Figures 3A–C, within pan-cancer tissues, the expression of FANCI exhibited a positive correlation with the majority of m1A, m5C, and m6A methylation modifications, underscoring FANCI’s potential role within the intricate regulatory network of mRNA modifications.

**FIGURE 4:**
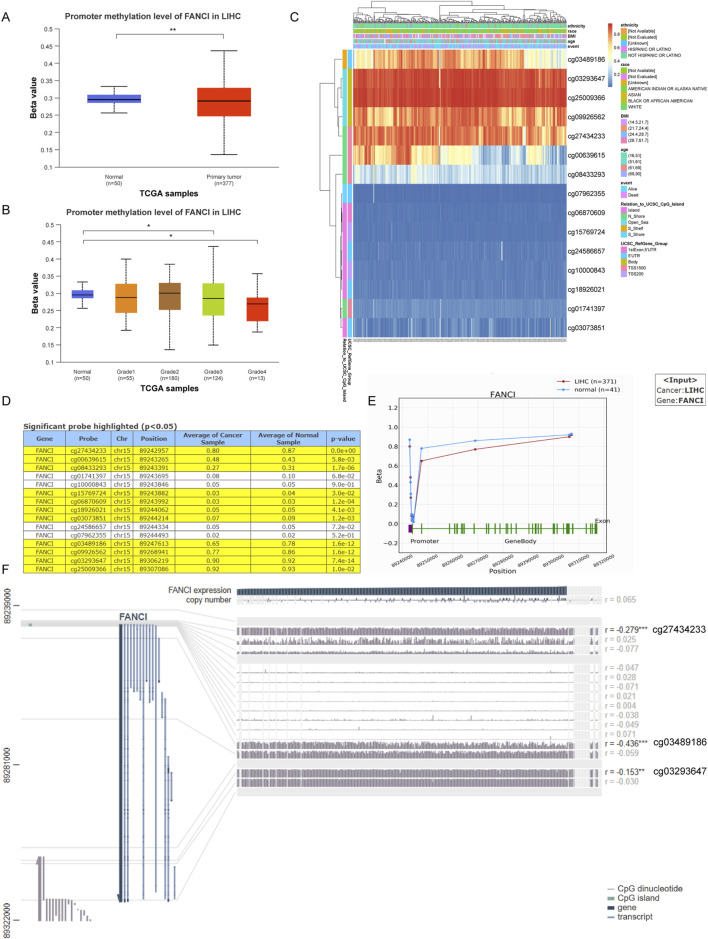
Analysis of FANCI Methylation in Liver Cancer. **(A,B)** Analysis of FANCI methylation in hepatic carcinoma and normal tissues utilizing the UALCAN database. **(C)** A heatmap depicting FANCI DNA methylation in LIHC, sourced from MethSurv. **(D,E)** Investigation of FANCI methylation status in LIHC was conducted using the OncoDB database. **(F)** MEXPRESS was employed to visualize methylation sites within the LIHC DNA sequence related to gene expression. FANCI expression is shown by the blue line. Pearson’s correlation coefficients and p-values for methylation sites and gene expression are indicated on the right. **P < 0.01, ***P < 0.001.

Finally, we used the UALCAN database to analyze the promoter methylation levels of FANCI in LIHC and normal tissues. Compared to normal tissues, the promoter methylation levels of FANCI were significantly reduced in primary liver tumors (Figure 4A, p < 0.01). In different tumor grades of LIHC, FANCI promoter methylation levels were significantly reduced, with lower methylation levels observed in higher-grade tumors (Figure 4B, p < 0.05). Figure 4C presents a heatmap of FANCI DNA methylation in LIHC from the MethSurv database. The heatmap reveals distinct methylation patterns at different CpG sites within the FANCI gene, with several sites showing differential methylation between tumor and normal tissues. Subsequently, the methylation status of FANCI in LIHC was explored in detail using the OncoDB database. Figure 4D lists probes with significant methylation differences, along with their respective locations, average β values in cancer and normal samples, and p-values. Several probes (e.g., cg27434233, cg03489186, cg03923647) exhibited significant differential methylation between LIHC and normal tissues (p < 0.05). Figure 4E visualizes methylation sites in the FANCI DNA sequence associated with gene expression. Finally, we used the MEXPRESS database to visualize methylation sites in the LIHC DNA sequence and their association with FANCI gene expression. Changes in methylation sites cg27434233 (R = −0.279), cg03489186 (R = −0.436), and cg03293647 (R = −0.153) were negatively correlated with FANCI expression (Figure 4F, P < 0.05). Overall, these results indicate that FANCI promoter methylation is significantly reduced in liver cancer compared to normal tissues, with specific methylation sites showing a strong correlation with FANCI expression. This highlights the potential role of FANCI methylation in the pathogenesis of liver cancer and its potential as a therapeutic target for LIHC gene therapy.

### Analysis of the correlation between FANCI expression and immune infiltration

**FIGURE 5:**
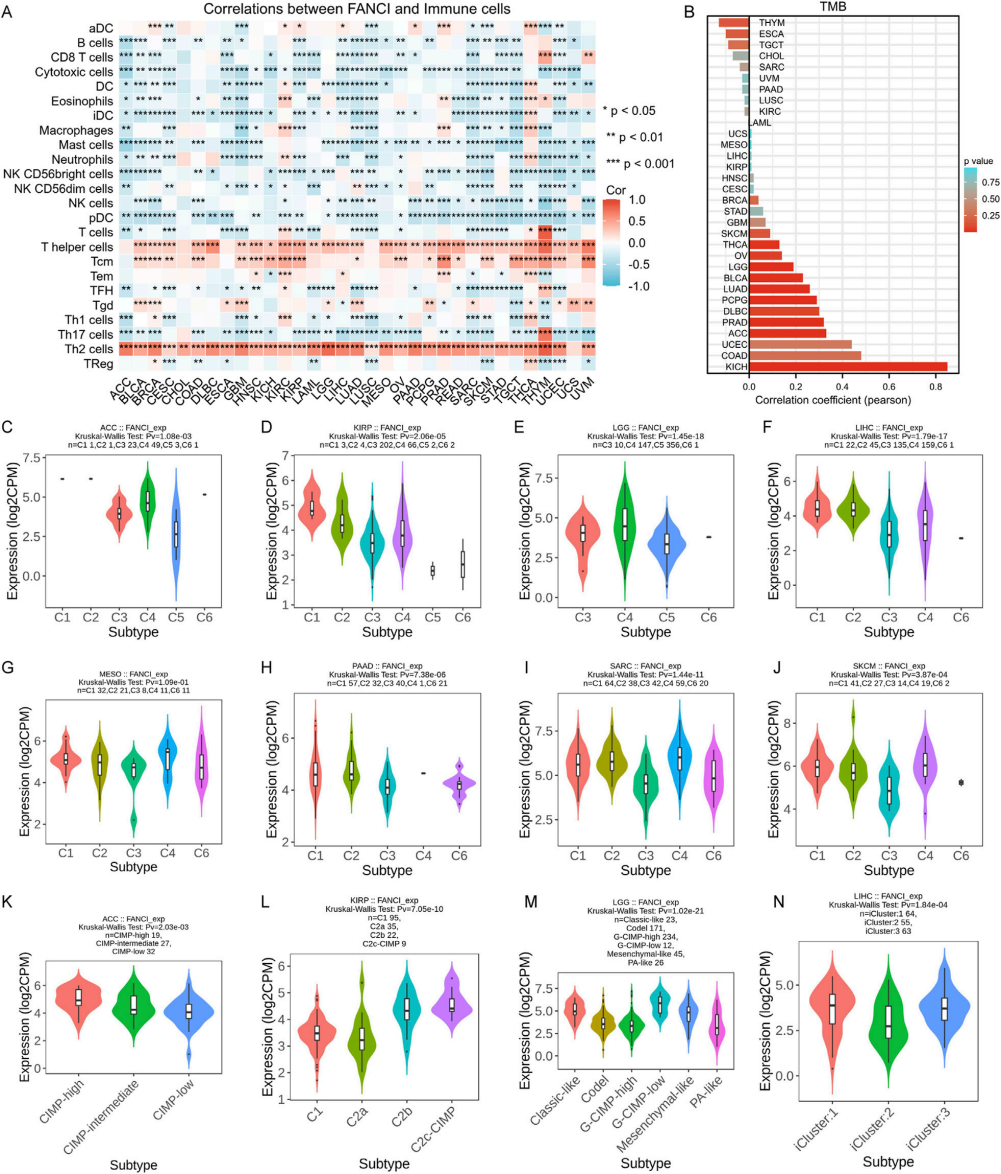
Correlation analysis of FANCI expression and immune infiltration. **(A)** FANCI expression and immune cell infiltration. **(B)** Correlation of FANCI expression and tumor mutation burden (TMB) in pan-cancer tissues. **(C–J)** Correlations between FANCI expression and immune subtypes in eight cancers. **(K–N)** Correlations between FANCI expression and molecular subtypes in four cancers. (*P < 0.05, **P < 0.01, ***P < 0.001).

We investigated the relationship between FANCI expression and immune cell infiltration in the tumor microenvironment (TME). Using the ssGSEA algorithm, the heat map showed a significant correlation between FANCI expression and the level of immune cell infiltration (Figure 5A). In various cancers, FANCI was positively correlated with T helper cells, Tcm, and Th2 cells. It was negatively correlated with the majority of immune cells such as Cytotoxic cells, NK cells, pDC, and Th17 cells. Subsequently, we explored the correlation between FANCI expression and tumor mutation burden (TMB) in pan-cancer tissues. The bar chart showed a significant positive correlation in several cancers, such as KICH, ACC, and PRAD, where higher FANCI expression was associated with increased TMB (Figure 5B). This suggests that FANCI expression may be related to genomic instability in these cancers. Figures 5C–J illustrate the correlation between FANCI expression and immune subtypes in eight cancers: ACC, KIRP, LGG, LIHC, MESO, PAAD, SARC, and SKCM. These violin plots show significant differences in FANCI expression among different immune subtypes. Figures 5K–N demonstrate the correlation between FANCI expression and molecular subtypes in four cancers: ACC, KIRP, LGG, and LIHC. These charts reveal significant differences in FANCI expression among different molecular subtypes, suggesting that FANCI expression may be associated with specific molecular characteristics of these cancers. Overall, these results emphasize the significant correlation of FANCI expression with immune infiltration, TMB, immune subtypes, and molecular subtypes in various cancers. This indicates that FANCI may play a role in regulating the tumor immune microenvironment and could serve as a potential biomarker for immune-related cancer characteristics.

### Molecular interaction network, functional enrichment, and transcriptional regulation analysis of FANCI

**FIGURE 6:**
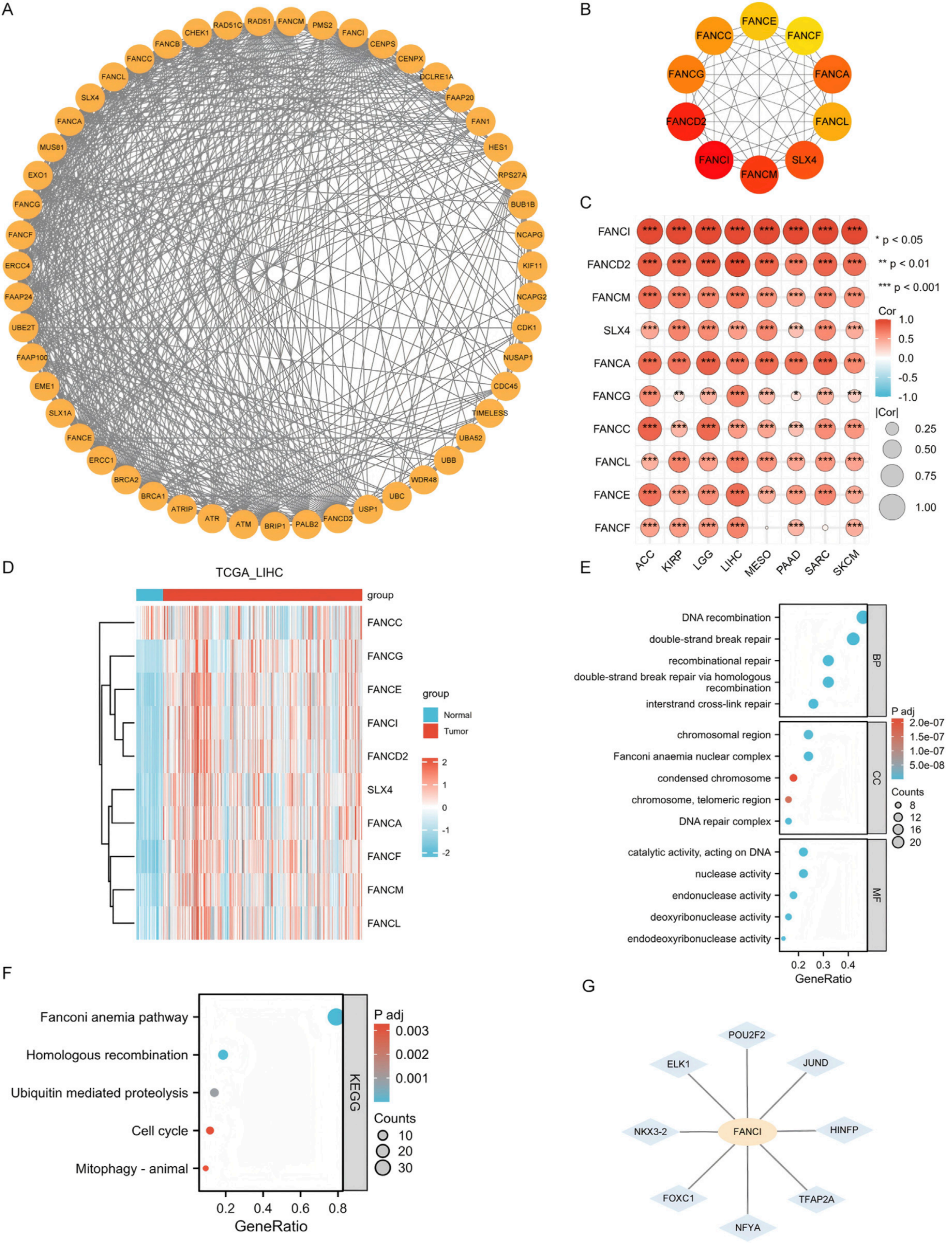
Integrated Analysis of FANCI’s Molecular Interaction Network, Functional Enrichment, and Transcriptional Regulation **(A)** The PPI network for FANCI. **(B)** The top ten hub genes within the PPI network. **(C)** A heatmap showing the association of hub genes with FANCI across eight cancers. **(D)** Heatmap showing the expression distribution of the top 10 core genes in LIHC tumor tissues and adjacent normal tissues. **(E,F)** GO and KEGG pathway enrichment for FANCI and closely interacting genes. **(G)** Predict the potential transcription factors of FANCI and screen out eight high-confidence regulatory factors: HINFP, POU2F2, ELK1, TFAP2A, NFYA, JUND, FOXC1, and NKX3-2. (*P < 0.05, **P < 0.01, ***P < 0.001).

A set of 50 genes closely associated with FANCI was identified using STRING, and a PPI network was constructed at the specified threshold (Figure 6A). The top 10 hub genes were FANCI, FANCD2, FANCM, SLX4, FANCA, FANCG, FANCC, FANCL, FANCE, and FANCF (Figure 6B). These hub genes are all significantly interconnected in cancers where FANCI expression influences prognosis (all p < 0.05) (Figure 6C).The heatmap shows that, compared to normal tissue, most of the top 10 hub genes have higher expression levels in tumor tissue (Figure 6D).

GO and KEGG enrichment analyses were conducted on these genes. RNA functions were categorized into biological process (BP), molecular function (MF), and cellular component (CC). The top GO terms for BP included DNA recombination, double-strand break repair, recombinational repair, homologous recombination, and interstrand cross-link repair. For CC, they were chromosomal region, Fanconi anemia nuclear complex, condensed chromosome, telomeric region, and DNA repair complex. For MF, they included catalytic activity on DNA, nuclease activity, endonuclease activity, deoxyribonuclease activity, and endodeoxyribonuclease activity (Figure 6E). The leading KEGG pathways were the Fanconi anemia pathway, homologous recombination, ubiquitin-mediated proteolysis, cell cycle, and mitophagy-animal (Figure 6F).

Additionally, to elucidate the transcriptional regulatory mechanism of FANCI, we utilized the NetworkAnalyst 3.0 platform to integrate the JASPAR 2022 database, predicting potential transcription factors in its promoter region (1,500 bp upstream of TSS). We identified eight high-confidence regulators: HINFP, POU2F2, ELK1, TFAP2A, NFYA, JUND, FOXC1, and NKX3-2 (Figure 6G). ELK1 and JUND regulate the cell cycle checkpoint pathway, aligning with the subsequent GSEA-enriched phenotypes of “DNA replication” and “cell cycle.” POU2F2 and NFYA are involved in the expression regulation of antigen-presenting genes (such as HLA-DRA), potentially mediating FANCI’s impact on immune infiltration. FOXC1 and NKX3-2 play crucial roles in the DNA damage response, complementing the functions of FANCI-interacting genes (FANCD2/FANCM).

**FIGURE 7:**
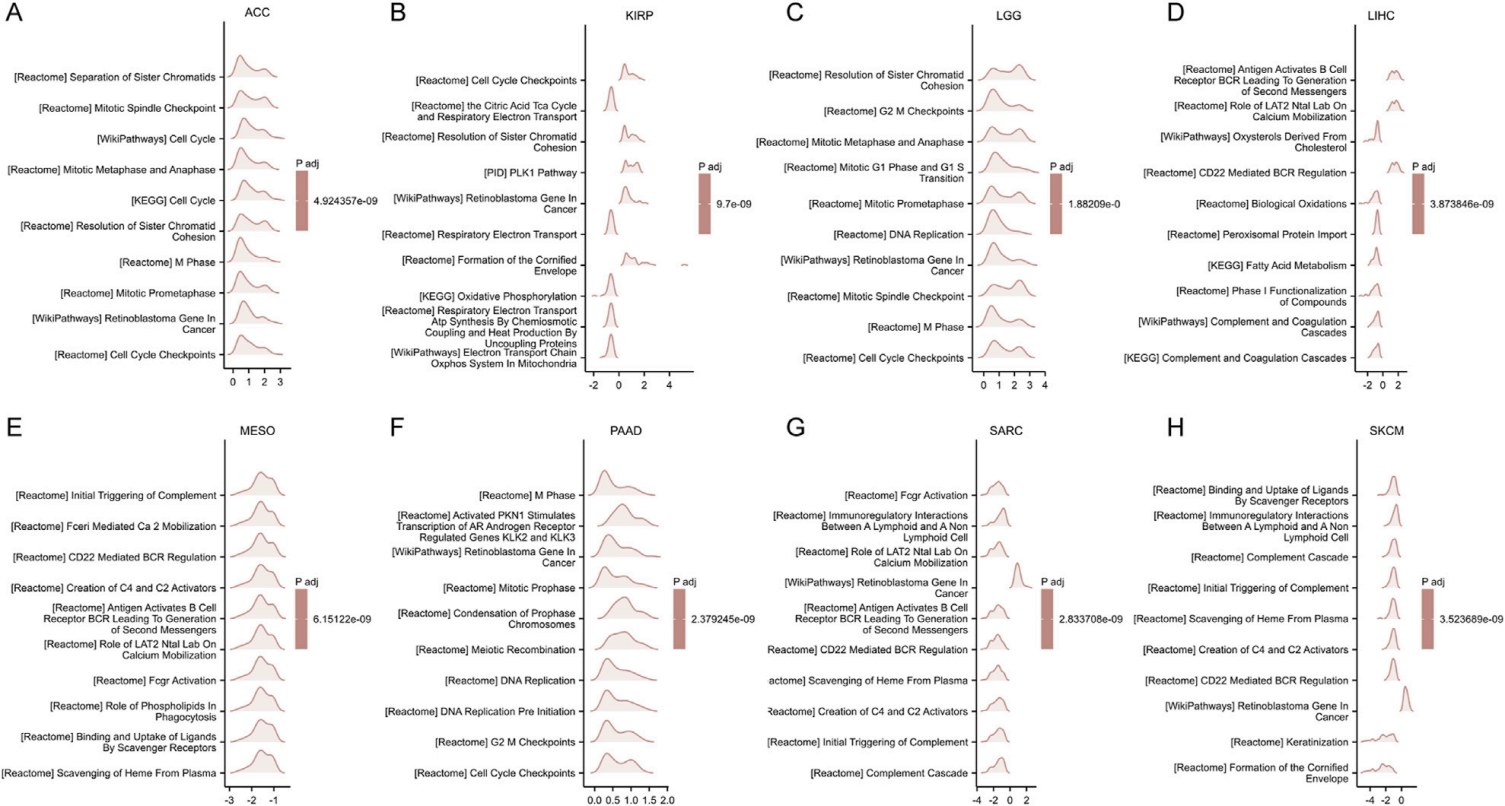
GSEA functional enrichment analysis of FANCI expression in 8 cancers. The top 10 GSEA functional enrichment pathways of FANCI in **(A)** ACC, **(B)** KIRP, **(C)** LGG, **(D)** LIHC, **(E)** MESO, **(F)** PAAD, **(G)** SARC, **(H)** SKCM. The Y-axis represents one gene set and the X-axis is the distribution of logFC corresponding to the core molecules in each gene set.

The GSEA results for eight types of cancer, where elevated FANCI expression predicts poor prognosis, are shown in Figures 7A–H. The commonly enriched pathways include cell cycle checkpoints, DNA replication, antigen presentation, and BCR signaling pathways. Overall, these GSEA results indicate that FANCI plays a significant role in several key pathways across different cancers, particularly those related to cell cycle regulation, immune response, and DNA replication.

### Functional states of FANCI in scRNA-Seq datasets

**FIGURE 8:**
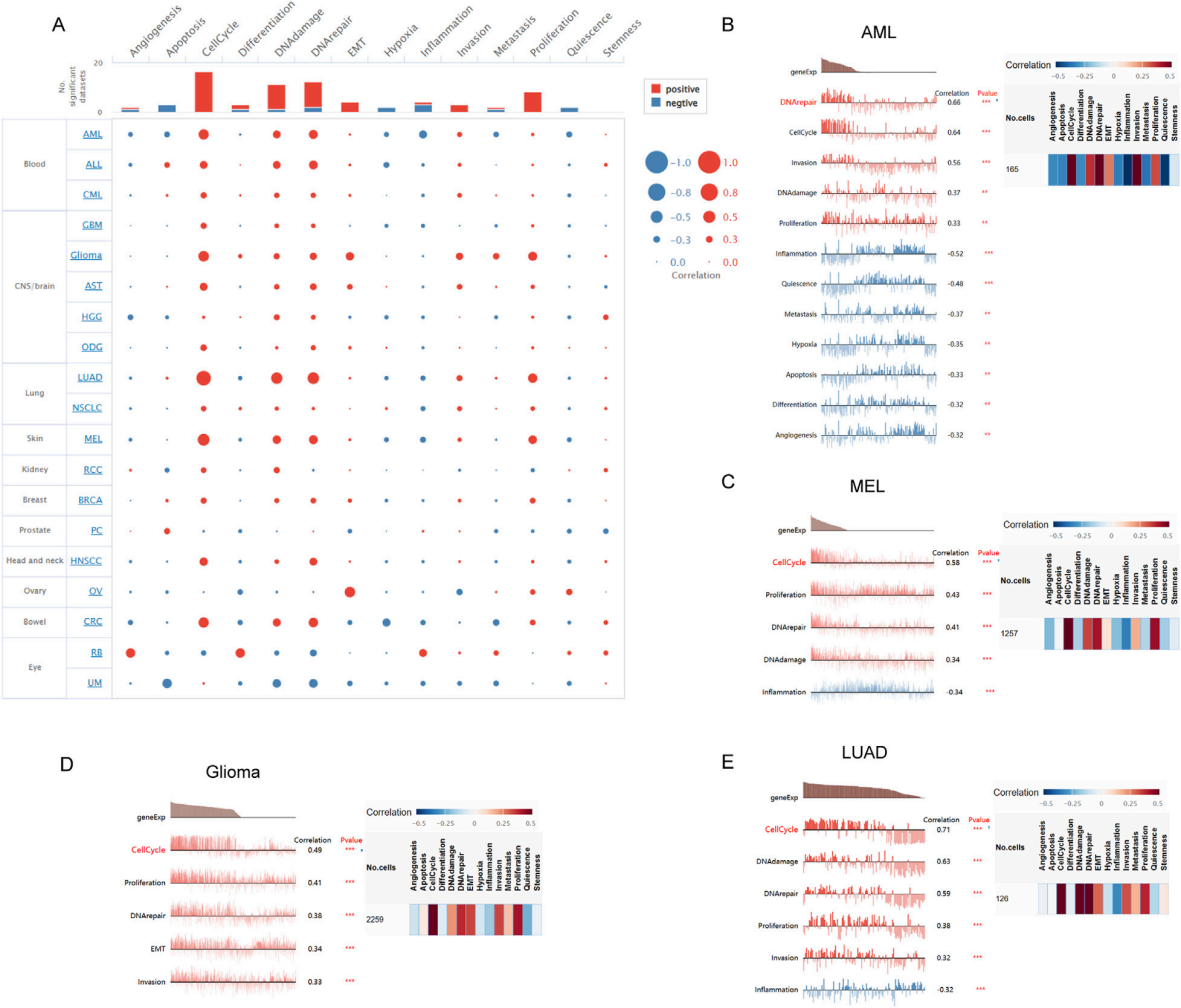
The correlation of FANCI with functional states in cancers is illustrated as follows: **(A)** An interactive bubble chart displaying the correlation of FANCI with functional states across 16 cancers. The correlation of FANCI with functional states in **(B)** AML, **(C)** MEL, **(D)** Glioma, and **(E)** LUAD. The X-axis represents different gene sets. (*P < 0.05, **P < 0.01, ***P < 0.001).

We investigated the functional role of FANCI across various cancer types using CancerSEA, which allows for the analysis of FANCI’s correlation with multiple functional states of cancer cells at the single-cell level. The findings indicated that FANCI expression positively correlates with cell cycle, DNA damage, DNA repair, EMT, invasion, and proliferation. Conversely, weak negative correlations were observed between FANCI expression and apoptosis, hypoxia, inflammation, and quiescence (Figure 8A). Subsequently, we examined the correlation between FANCI and functional status in specific cancers.The study demonstrated that in acute myeloid leukemia (AML), FANCI exhibits a positive correlation with processes such as DNA repair, cell cycle progression, cellular invasion, DNA damage response, and cellular proliferation. Conversely, it shows a negative correlation with inflammation, cellular quiescence, metastasis, hypoxic conditions, apoptosis, cellular differentiation, and angiogenesis. In the context of melanoma (MEL), FANCI is positively associated with cell cycle regulation, cellular proliferation, DNA repair mechanisms, and DNA damage, while it is inversely associated with inflammatory responses. In glioma, FANCI is positively linked to cell cycle activities, cellular proliferation, DNA repair, epithelial-mesenchymal transition (EMT), and invasive behavior. Regarding lung adenocarcinoma (LUAD), FANCI demonstrates positive correlations with cell cycle regulation, DNA damage response, DNA repair, cellular proliferation, and invasion, and a negative correlation with inflammation(Figures 8B–E).

### Establishment of a ceRNA network for LIHC

**FIGURE 9:**
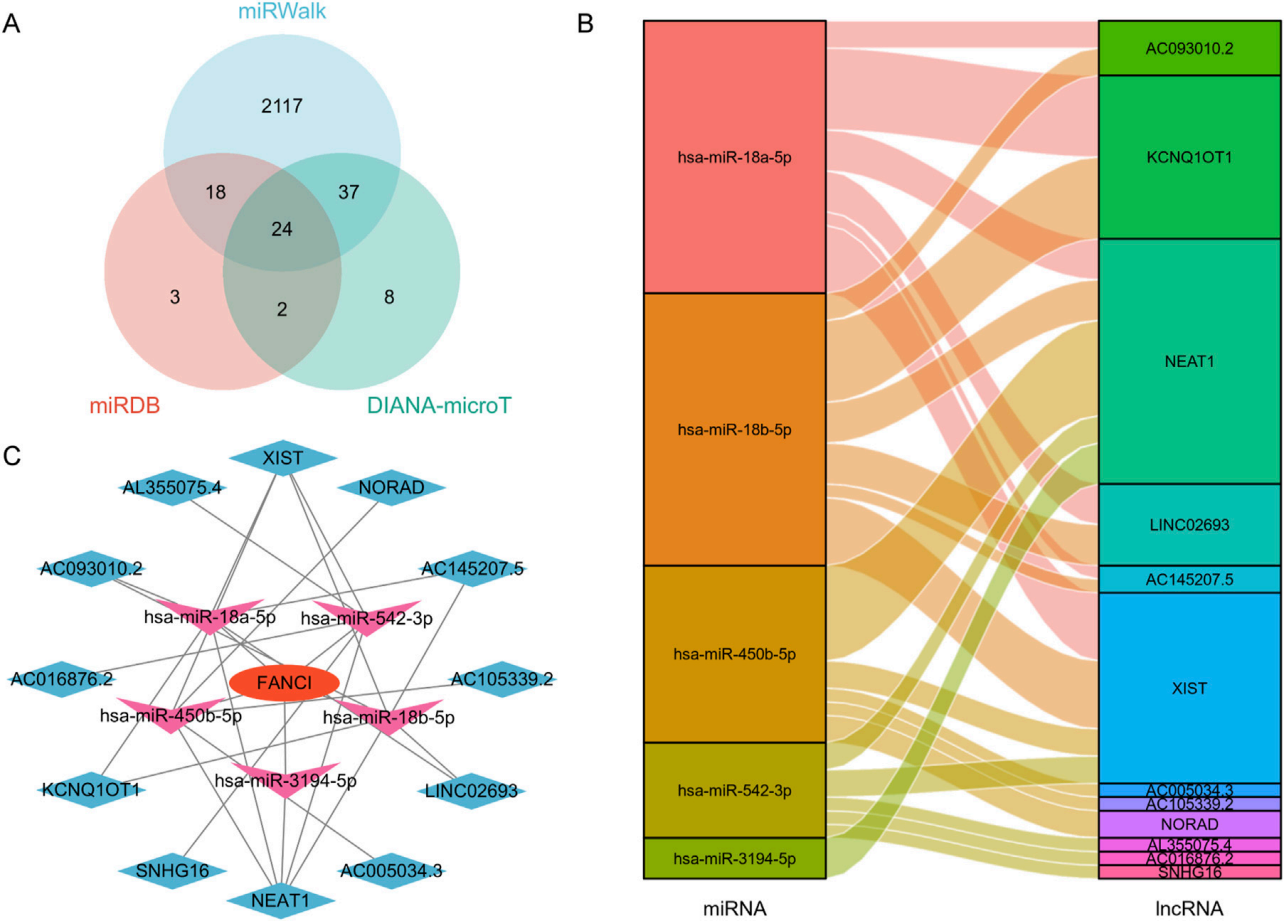
Construction of a FANCI-associated ceRNA regulatory network in LIHC. **(A)** Venn diagram illustrating the overlap of FANCI-targeted miRNAs predicted by DIANA-microT, miRWalk, and miRcode. **(B)** Sankey diagram depicting the relationship between target miRNAs and their corresponding target lncRNAs. **(C)** A lncRNA-miRNA-FANCI interaction network was established for LIHC using Cytoscape.

Figure 9A presents a Venn diagram showing the overlap of predicted FANCI target miRNAs from three different databases: DIANA-microT, miRWalk, and miRcode. The Venn diagram reveals 24 common miRNAs predicted by all three databases. Additionally, there are miRNAs predicted by combinations of two databases or uniquely by one database. This overlap indicates a robust set of miRNAs likely to interact with FANCI in LIHC.

Figure 9B shows a Sankey diagram illustrating the relationships between the target miRNAs and their corresponding target long non-coding RNAs (lncRNAs). Key findings include miRNAs, such as hsa-miR-18a-5p, hsa-miR-18b-5p, and hsa-miR-450b-5p, that are connected to multiple lncRNAs. lncRNAs like NEAT1, XIST, and NORAD are targeted by several miRNAs. This diagram highlights the complex interactions between miRNAs and lncRNAs in the context of FANCI regulation within the lncRNA-miRNA-FANCI Regulatory Network.

Figure 9C presents a network diagram constructed using Cytoscape, showing the lncRNA-miRNA-FANCI regulatory network in LIHC. This network illustrates the regulatory complexity involving FANCI, miRNAs, and lncRNAs, suggesting that FANCI expression in LIHC is modulated by a competing endogenous RNA (ceRNA) network. Overall, these results demonstrate the construction of a comprehensive ceRNA network involving FANCI in LIHC. The identification of key miRNAs and lncRNAs interacting with FANCI highlights potential regulatory mechanisms and provides insights into the molecular interactions that may influence FANCI expression and function in liver cancer.

### Knockdown of FANCI suppresses migration and tumor invasion in LIHC cells

**FIGURE 10:**
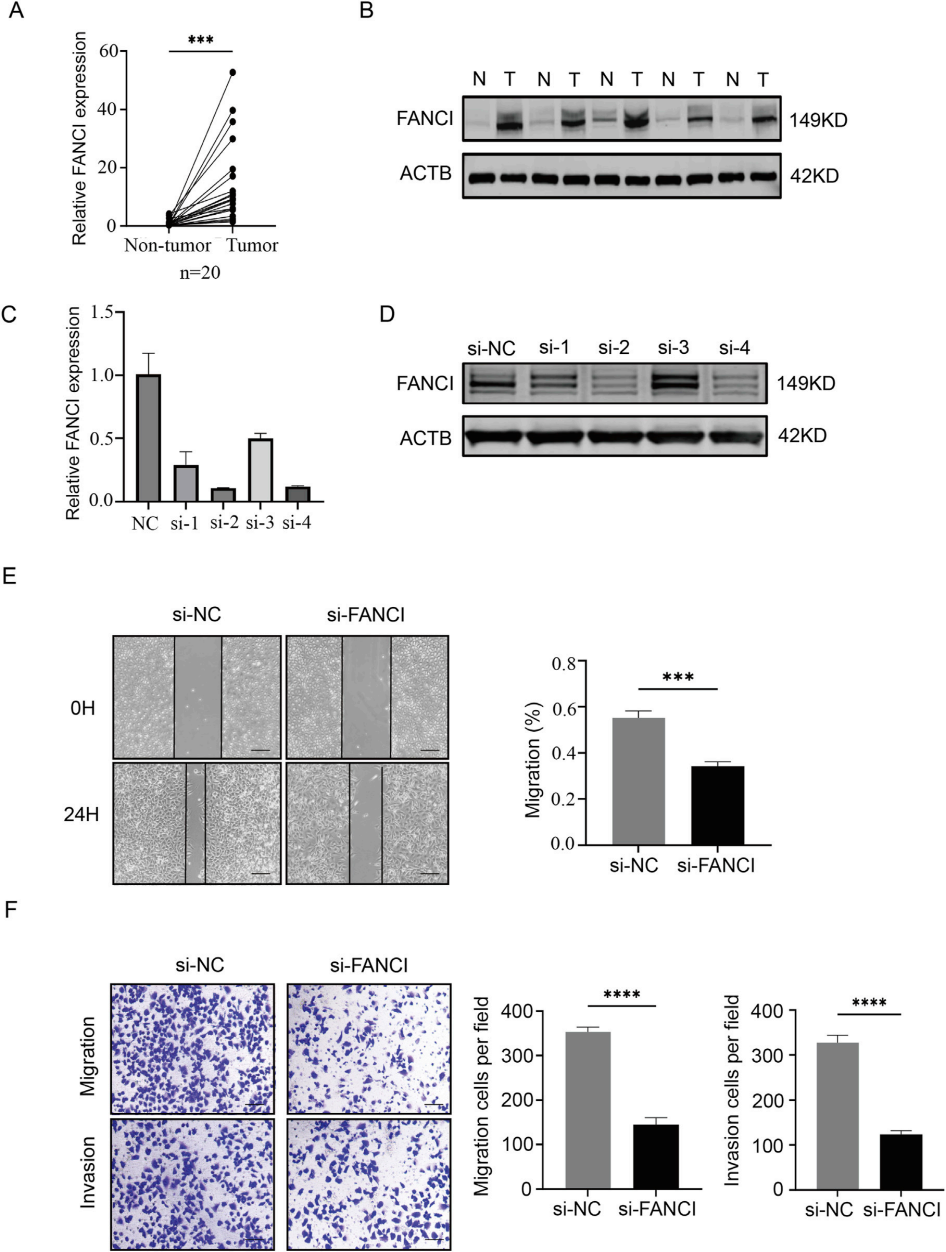
Knockdown of FANCI suppresses cell migration and invasion. **(A,B)** Western blot and qPCR were performed to detect the expression level of FANCI in LIHC tissues. **(C,D)** Quantitative analysis of FANCI mRNA and protein in Huh7 cells following treatment with FANCI siRNA to verify the silencing efficiency of different siRNAs. **(E,F)** Wound healing assay and Transwell migration assay were used to evaluate the impact of FANCI downregulation on the migratory or invasive abilities of Huh7 cells. Scale bars, 100 × = 400 μm. All experiments were implemented three times, and data are presented as mean ± SD.

We examined the expression of FANCI in LIHC, and our results revealed that FANCI mRNA and protein levels were significantly higher in tumor tissues than in the corresponding non-tumor tissues (Figures 10A,B). To further investigate the role of FANCI in LIHC, we conducted experiments using Huh7 cells. In our study, following siRNA infection (Figures 10C,D), the wound healing assay results indicated that the migration speed slowed after FANCI was knocked down (Figure 10E). Similarly, the transwell results showed that the migration and invasion abilities of the cells were reduced after FANCI was knocked down (Figure 10F). These experimental results support the role of FANCI as a pivotal oncogene that facilitates LIHC cell invasion and migration.

## Discussion

This study integrates multi-omics data to systematically investigate the expression profile, prognostic significance, diagnostic performance, and associations of FANCI with genetic alterations, epigenetic regulation, the tumor immune microenvironment, and key signaling pathways across multiple cancers. Preliminary experiments further validated the expression levels and biological functions of FANCI in LIHC. The findings reveal that FANCI is significantly upregulated in most cancer types and closely associated with poor outcomes, suggesting that it plays a key oncogenic role in tumor initiation and progression.

FANCI is a critical component of the Fanconi anemia pathway. Its primary function is to form a heterodimeric complex with FANCD2; following DNA damage, FANCI becomes monoubiquitinated, which recruits DNA repair proteins to the site of damage and initiates the repair of DNA interstrand crosslinks (Smogorzewska et al., 2007; Sareen et al., 2012; Koczorowska et al., 2014; Rego et al., 2012a; Lopez-Martinez et al., 2019). Our results indicate that FANCI is significantly overexpressed in most cancer types, implying that by enhancing DNA damage repair capabilities, it may help cancer cells adapt to high levels of DNA damage stress, thereby promoting oncogenesis and tumor progression.

By integrating data from the TCGA and GTEx databases, we systematically analyzed the expression pattern and clinical relevance of FANCI in 33 cancer types. FANCI was found to be upregulated in multiple tumors, consistent with previous findings in CESC (Liu et al., 2021), BRCA (Huang et al., 2024), LUAD (Li et al., 2023), and SKCM (Cai et al., 2023). Moreover, FANCI expression was closely linked to tumor staging and grading, particularly in LIHC, where its expression significantly increased with tumor progression, suggesting a critical role in liver cancer development. Further Cox regression and Kaplan–Meier survival analyses demonstrate that high FANCI expression is significantly associated with poor prognosis in cancers such as KIRP, LIHC, PAAD, and ACC. Additionally, ROC curve analyses confirm that FANCI holds high diagnostic value in LIHC and PAAD (AUC >0.9), indicating its potential both as a prognostic biomarker and as an effective diagnostic indicator.

Our analysis also revealed that FANCI expression is subject to precise, multidimensional regulation. In LIHC, hypomethylation of the FANCI promoter is significantly negatively correlated with its overexpression, suggesting that epigenetic dysregulation is one of the key mechanisms driving aberrant FANCI expression in liver cancer. Moreover, bioinformatic predictions identified several potential transcriptional regulators of FANCI—such as HINFP, POU2F2, ELK1, JUND, and FOXC1. Previous studies have demonstrated that these transcription factors play critical roles in various cancers; for example, HINFP shows significant regulatory effects in bladder cancer (Zheng et al., 2022), POU2F2 is linked to lung cancer (Luo et al., 2021), and JUND and FOXC1 are key in colorectal (Chang et al., 2023) and breast cancer (Sizemore and Keri, 2012), respectively. They may act in concert to promote FANCI overexpression in diverse cancer contexts. In addition, we constructed a lncRNA-miRNA-FANCI competing endogenous RNA regulatory network in LIHC, revealing the complexity of posttranscriptional regulation and offering new insights into the mechanisms of FANCI dysregulation.

Functional enrichment analyses (GO, KEGG, GSEA) and single-cell functional state analysis (CancerSEA) consistently show that FANCI-associated gene sets are primarily enriched in core biological processes such as DNA damage repair, DNA replication, cell cycle regulation, and homologous recombination. This is highly consistent with FANCI’s known role in the Fanconi anemia pathway (Smogorzewska et al., 2007; Longerich et al., 2014; Niraj et al., 2019). Notably, in cancers where high FANCI expression is linked to poor prognosis, pathways related to cell cycle checkpoints, DNA replication, and antigen processing and presentation are significantly enriched. These findings offer clues on how FANCI could promote tumor progression by maintaining genomic stability and supporting cell proliferation, while also possibly affecting tumor cell immunogenicity. Based on these analyses, we speculate that high FANCI expression not only facilitates DNA repair but also represents an adaptive mechanism—enabling cancer cells to tolerate higher levels of genomic instability and avoid lethal damage, thereby securing a survival advantage in rapidly proliferating and hostile microenvironments (Fierheller et al., 2021; Rego et al., 2012b; Hu et al., 2019). Specifically, FANCI-mediated DNA damage response may synergize with aberrant cell cycle checkpoint regulation (as indicated by GSEA-enriched pathways), allowing damaged cells to continue dividing and accelerating tumor heterogeneity and evolution (Maleki et al., 2024). Moreover, its roles in DNA replication and homologous recombination could directly enhance cancer cell capacity to maintain high replication rates and cope with replicative stress.

In addition, our analysis shows that FANCI expression is positively correlated with the infiltration of various immune cells—such as helper T cells, central memory T cells, and Th2 cells—while it is negatively correlated with the infiltration of cytotoxic T cells, NK cells, and plasmacytoid dendritic cells, as well as with the tumor mutational burden in certain cancers. These findings are consistent with previous studies on the relationship between FANCI and immune infiltration in cervical cancer (Liu et al., 2021), melanoma (Cai et al., 2023), liver cancer (Hou et al., 2023), and lung adenocarcinoma (Ye et al., 2021). Such correlations suggest that FANCI may play a role in modulating the tumor immune microenvironment, thereby influencing immune evasion or immune response. In light of the enrichment of antigen processing pathways identified through GSEA analysis, we speculate that dysregulation of FANCI might reduce the efficiency of tumor antigen presentation, promoting immune evasion. Its regulation of DNA damage repair could also indirectly affect genomic stability and neoantigen production, thereby altering the recruitment and activation of immune cells. The observed pattern of increased immunosuppressive cells coupled with a decrease in effector cells may hint at direct or indirect signaling crosstalk. Clarifying these association-based hypotheses is critical for developing effective combination treatment strategies, such as pairing immunotherapy with DDR inhibitors (Chan et al., 2019; Alhmoud et al., 2020; Sun et al., 2023). Future studies could explore whether FANCI expression levels might serve as a potential predictive marker for immunotherapy responses.

To validate predictions from our bioinformatics analysis, we performed functional experiments in LIHC tissues and cell lines. Results revealed that FANCI is highly expressed in liver cancer tissues, and specific downregulation of FANCI using siRNA significantly inhibited cellular migration and invasion, as demonstrated in wound-healing and Transwell assays. These findings directly confirm that FANCI promotes the malignant phenotype of LIHC cells *in vitro*, thereby providing preliminary experimental evidence for its potential as a therapeutic target.

This study reveals the diagnostic and prognostic value of FANCI across multiple cancers, particularly demonstrating exceptionally high diagnostic accuracy (AUC >0.9) in LIHC and PAAD. These results suggest that FANCI has the potential to serve as an effective biomarker for early diagnosis and prognostic evaluation in liver and pancreatic cancers. Clinically, assessing FANCI expression in patient tissues or peripheral blood could aid in early tumor diagnosis and risk stratification. Moreover, the close association between FANCI expression with tumor staging and pathological grading further indicates its potential utility for monitoring tumor progression and evaluating therapeutic outcomes. Regarding treatment, given FANCI’s key role in DNA damage repair within tumor cells, targeting FANCI may enhance tumor cell sensitivity to radiotherapy and chemotherapy, thereby improving treatment efficacy. Our cell experiments also demonstrate that knocking down FANCI significantly suppresses the migration and invasion capabilities of liver cancer cells. Therefore, developing specific small-molecule inhibitors or RNA interference drugs targeting FANCI could represent a novel therapeutic strategy against malignancies such as liver cancer. In addition, considering the close relationship between FANCI and the tumor immune microenvironment, combining FANCI inhibitors with immune checkpoint inhibitors might further enhance the efficacy of tumor immunotherapy.

Although this study provides comprehensive bioinformatics insights and is supported by preliminary experimental validation, it primarily relies on public databases and computational analyses, with relatively limited *in vitro* experiments; this is the main limitation of the work. Future research urgently needs to adopt more complex model systems (such as gene-edited cell lines, tumor organoids, and patient-derived xenograft models) and advanced techniques (such as proteomics and single-cell sequencing) to precisely delineate the specific molecular pathways and interaction networks through which FANCI regulates DNA damage response, the cell cycle, and immune signaling in different cancer contexts. Concurrently, systematic screening and optimization of inhibitors targeting FANCI (or its pathways) should be conducted, with their efficacy and safety evaluated in preclinical models. Moreover, large-scale, multicenter prospective clinical cohort studies should be initiated to establish standardized FANCI detection methods and to confirm the association between its expression levels and responses to specific treatments (chemotherapy, targeted therapy, immunotherapy), which is a key step in exploring its clinical value as a predictive biomarker.

In summary, this study, through an integration of multi-omics analyses and preliminary experimental validation, systematically delineates the expression patterns, clinical significance, and potential functions of FANCI across cancers. The research emphasizes the ubiquitous upregulation of FANCI and its importance as both an adverse prognostic marker and a potential diagnostic biomarker in various cancers. Functional analyses and mechanistic investigations reveal that FANCI plays a critical role in maintaining genomic stability, driving cell proliferation, and shaping the tumor immune microenvironment, with its expression being intricately regulated at the epigenetic, transcriptional, and post-transcriptional levels. Preliminary *in vitro* experiments have confirmed its role in promoting invasion and migration in LIHC. These findings not only deepen our understanding of the complex role of FANCI in cancer biology but also lay the groundwork for further development of FANCI as a potential therapeutic target and biomarker, thereby paving the way for future research aimed at advancing personalized cancer treatment strategies.

## Data Availability

The original contributions presented in the study are included in the article/Supplementary Material, further inquiries can be directed to the corresponding author.
