# Supplementary figures and images for: Expression and clinical significance of FANCI gene in pan-cancer: a comprehensive analysis based on multi-omics data
Source: Front Genet. 2025 May 9;16:1542888. doi: 10.3389/fgene.2025.1542888 (PMC12098372; doi:10.3389/fgene.2025.1542888)

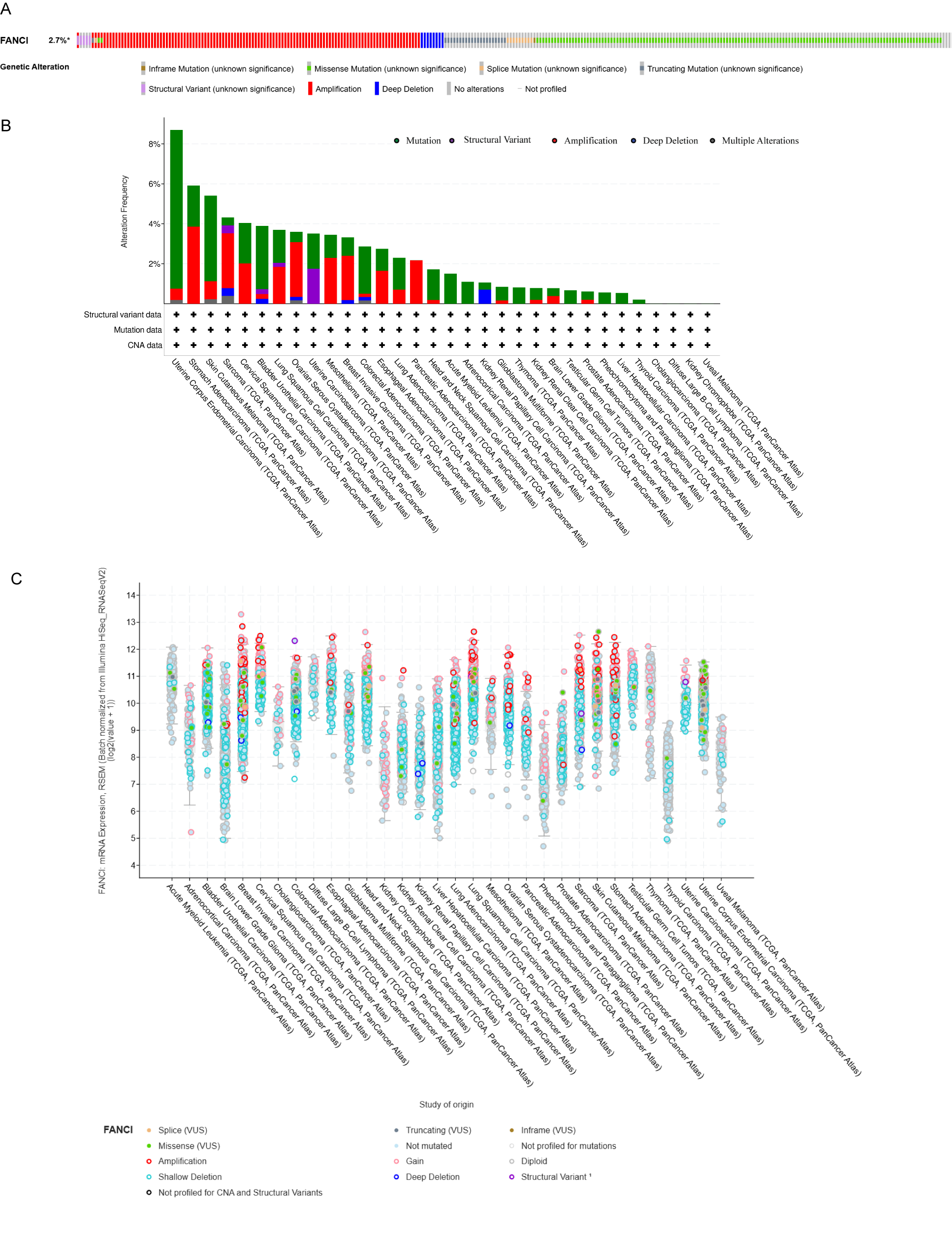

Supplement: Supplementary file 1 [file Image1.TIF]
